# PyMT-Maclow: A novel, inducible, murine model for determining the role of CD68 positive cells in breast tumor development

**DOI:** 10.1371/journal.pone.0188591

**Published:** 2017-12-08

**Authors:** Robin M. H. Rumney, Seth B. Coffelt, Terence A. Neale, Sandeep Dhayade, Gillian M. Tozer, Gaynor Miller

**Affiliations:** 1 Department of Oncology and Metabolism, University of Sheffield, Sheffield, United Kingdom; 2 Department of Infection & Immunity, University of Sheffield, Sheffield, United Kingdom; 3 University of Glasgow, Institute of Cancer Sciences, Glasgow, United Kingdom; 4 Cancer Research UK Beatson Institute, Glasgow, United Kingdom; University of Tennessee Health Science Center, UNITED STATES

## Abstract

CD68^+^ tumor-associated macrophages (TAMs) are pro-tumorigenic, pro-angiogenic and are associated with decreased survival rates in patients with cancer, including breast cancer. Non-specific models of macrophage ablation reduce the number of TAMs and limit the development of mammary tumors. However, the lack of specificity and side effects associated with these models compromise their reliability. We hypothesized that specific and controlled macrophage depletion would provide precise data on the effects of reducing TAM numbers on tumor development. In this study, the MacLow mouse model of doxycycline-inducible and selective CD68^+^ macrophage depletion was crossed with the murine mammary tumor virus (MMTV)-Polyoma virus middle T antigen (PyMT) mouse model of spontaneous ductal breast adenocarcinoma to generate the PyMT-MacLow line. In doxycycline-treated PyMT-MacLow mice, macrophage numbers were decreased in areas surrounding tumors by 43%. Reducing the number of macrophages by this level delayed tumor progression, generated less proliferative tumors, decreased the vascularization of carcinomas and down-regulated the expression of many pro-angiogenic genes. These results demonstrate that depleting CD68^+^ macrophages in an inducible and selective manner delays the development of mammary tumors and that the PyMT-MacLow model is a useful and unique tool for studying the role of TAMs in breast cancer.

## Introduction

Macrophages are extremely versatile cells found in every tissue of the body, whose functions are often required for organism development, maintenance of tissue homeostasis and immunity [1]. Macrophages are also important in cancer biology, where they are referred to as tumor-associated macrophages (TAMs). In the majority of human cancers, high numbers of TAMs or enrichment of TAM-associated gene signatures are correlated with poor prognosis [2, 3]. TAMs can influence tumor progression both negatively and positively by secreting cytotoxic factors or stimulating cancer cell proliferation, angiogenesis, immunosuppression, invasion and metastasis to distant organs [2–4]. Moreover, several experimental studies have now shown that the efficacy of anti-cancer therapies is largely determined by macrophage function [3, 5, 6]. In light of these data, TAM inhibitors–such as those that interfere with CSF1R signalling [7]–are currently being tested in combination with chemotherapy, radiotherapy, angiogenesis inhibitors and/or T cell checkpoint inhibitors in clinical trials. The outcome of most of these trials have yet to be reported [8].

To establish the importance and varied roles of TAMs in regulating tumor progression and metastasis, many targeting approaches have been investigated in various mouse models of cancer. Seminal studies showed that ablating macrophages genetically by deleting the colony-stimulating factor 1 gene, *Csf1*, in a transgenic mouse model of breast adenocarcinoma delays tumor development and the angiogenic switch to prevent metastasis [9, 10]. The use of other strategies, such as administration of an attenuated strain of the bacteria *Shigella flexneri* [11], a DNA minigene vaccine [12], siRNAs and liposome encapsulated chlodronate [13], cause tumor regression and/or reduced angiogenesis in murine tumor models. However, these approaches all have their limitations: namely, poor efficiency, lack of specificity, duration of macrophage depletion and the induction of detrimental side effects such as substantial toxicity and reduced immunity.

Previously, we generated a novel mouse model, called MacLow, for the doxycycline-inducible depletion of CD68^+^ macrophages *in vivo* [14]. Administering doxycycline either by intraperitoneal injection or in the animals’ chow results in a depletion of up to 50% of the tissue resident macrophages in the liver, spleen and bone. Importantly, the inducibility of this model circumvents the negative effects of macrophage depletion on embryonic and reproductive organ development. Peritoneal macrophages taken from doxycycline treated animals are also functionally impaired as demonstrated by their reduced ability to mount a cytokine response to LPS stimulation [14]. In the current study, we utilize this model to determine the effects of CD68^+^ macrophage depletion on tumor development in the murine mammary tumor virus polyoma middle T antigen (PyMT) transgenic mouse model of spontaneous breast ductal adenocarcinoma [15]. The PyMT mouse model has clearly definable stages of disease that correlate with similar stages in human breast cancer [16]. We crossed MMTV-PyMT mice with MacLow mice to generate PyMT-MacLow mice. Treating PyMT-MacLow animals with doxycycline induced depletion of tissue resident macrophages in the liver by ~40%, as previously published [14]. Moreover, TAMs surrounding tumors were reduced by ~43%. The decrease in TAMs was associated with a delay in tumor progression, a lower proliferative index and reduced microvessel density. Thus, the PyMT-MacLow model represents a new, inducible tool to study the role of TAMs in tumor progression, metastasis and anti-cancer therapy response.

## Materials and methods

### Animal maintenance and treatment

All experiments were conducted in accordance with the United Kingdom Animals (Scientific Procedures) Act 1986, with local ethical approval from the University of Sheffield Animal Welfare and Ethical Review Panel under the authority of a UK Home Office Project Licence (PPL 40/3125). Mice were treated with doxycycline at 3 weeks of age for 7 weeks by allocating 10g of food containing 625mg/kg of doxycycline (Harlan Laboratories Inc, Madison, USA) to each mouse. The chow containing doxycycline was replaced with fresh food every other day to ensure that the activity of the doxycycline was not compromised by prolonged exposure to light and heat. Water was provided *ad libitum* (S1 ARRIVE Checklist). Animals were checked daily and all efforts were made to minimise animal suffering. Animals were euthanised by cervical dislocation when primary tumor burden reached 12.5 mm mean diameter or when the tumor restrained animal agility. No other adverse effects were observed during the study and no animals died before meeting this humane endpoint.

### Generation of the inducible macrophage depletion model of breast cancer (PyMT-MacLow)

The inducible macrophage depletion line, MacLow, generated as described previously [14], contains two transgenes (CD68 and tetDTA) and is on an FVB/n background. Female MacLow animals were crossed with male MMTV-PyMT animals [15] on an FVB/n background (kind gift from Professor Nicola Brown, University of Sheffield) to generate triple transgenic (PyMT-MacLow) animals that were heterozygous for all three transgenes. Female PyMT-MacLow animals were used in future studies alongside female littermates that were heterozygous for the MMTV-PyMT and tetDTA (designated as PyMT) or the CD68 and tetDTA transgenes (designated as MacLow) as controls. Animals were genotyped by a PCR which amplified regions in the CD68 and tetDTA transgenes as previously described [14] and a 556 bp region of the PyMT transgene using primer pair 5′-GGAAGCAAGTACTTCACAAGGG-3′ and 5′- GGAAAGTCACTAGGAGCAGGG-3′. A control PCR was also performed which amplified 324 bp product using primer pair 5′-CTAGGCCACAGAATTGAAAGATCT-3′ and 5′- GTAGGTGGAAATTCTAGCATCATCC-3′. Reactions were heated to 94°C for an initial 5 min and then amplified by denaturing at 94°C for 30 sec, annealing at 55°C for 30 sec and extending at 72°C for 1 min, for a total of 35 cycles. Products were visualised by agarose gel electrophoresis. The following controls were used: age and sex matched PyMT-MacLow animals fed with normal chow; MacLow and PyMT littermate animals (see above) fed normal chow and doxycycline containing chow. 5–7 animals of each genotype were randomly assigned to each treatment group and were analysed blinded to treatment group and genotype.

### Flow cytometry

Tumors were collected from four 10-week-old MMTV-PyMT mice (kind gift from Karen Blyth, CRUK Beatson Institute) and disassociated with collagenase A and DNAse as previously described [17]. Single cell suspensions were stained with anti-CD45-PE-Cy7 (1:100), anti-CD11b-FITC (1:400), anti-F4/80-PerCP (1:200) and anti-CSF1R-PE (1:200). Cells were fixed and permeabilized using the Cytofix/Cytoperm™ kit (BD Biosciences) and followed by intracellular staining wtih anti-CD68-APC (1:200). All antibodies were purchased from eBioscience. This experiment was performed using a BD Fortessa flow cytometer using Diva software. Data analyses were performed using FlowJo Software version 9.9.4.

### PCR array

Using an RNeasy kit (Qiagen) total RNA was isolated from 30 mg pieces of mammary fat pad containing tumours obtained from three control and three doxycycline treated PyMT-MacLow animals. Samples of RNA were subjected to an on column DNAse digestion according to manufacturers instructions and an additional DNAse treatment was performed post elution using RQ1 DNase (Promega). cDNA was synthesised using Superscript III reverse transcriptase (ThermoFisher Scientific, Paisley, UK) and oligoDT primers following the manufacturers instructions. The three samples of cDNA were pooled for control and doxycycline treated groups to give an overall concentration of 70ng/μl. Gene expression levels were analysed by hybridising 9.24μg of pooled cDNA, mixed with SYBR green mastermix (Qiagen) to two Mouse Angiogenesis RT^2^ (Qiagen) plates (control and treated) under the following conditions: 10 minutes at 95°C, followed by 40 cycles of 15 seconds at 95°C and 1 minute at 60°C. The microarray was conducted in an Applied Biosystems 7900 thermocycler and the data generated in SDS 2.3 that collected and interpreted the data, the baseline and threshold values were set to automatic. Genes exhibiting a three-fold or greater change were considered biologically relevant.

### Histology and immunohistochemistry

The entire mammary fat pad and a sample of liver was excised from all animals at the end of the treatment period. Tissue was fixed in 10% formalin and paraffin embedded. 5 μm sections from each formalin-fixed paraffin-embedded tissue were stained with haematoxylin and eosin (H&E). Antigen retrieval specific to each antibody was performed as follows: by incubation with trypsin (anti-F4/80); by heating in 0.05% tween in Tris-EDTA buffer (anti-Ki67) or by incubation in antigen retrieval buffer (Dako, anti-CD31) [18]. Blocking was performed with rabbit or goat serum and slides were incubated with the primary antibody for one hour at room temperature [anti-F4/80 Clone C1:A3-1 (abcam, Cambridge, UK) 1:50 dilution, anti-Ki67 (abcam) 1:1000 dilution] or overnight at 4°C [anti-CD31 (Dianova GmbH, Hamburg, Germany) 1:200 dilution] [18]. Sections were incubated with secondary antibodies raised to the appropriate species using the Vectastain ABC kit (Vector Laboratories Ltd, Peterborough, UK) followed by DAB chromogenic detection.

### Analysis and quantification

The number of macrophages in the liver was determined by counting F4/80 positive cells in five randomly selected fields of view for each tissue section using a 20x objective.

Mammary sections labelled for F4/80 were scanned with an Aperio ScanScope Model CS slide scanner (Aperio Inc Vista CA USA). Five randomly selected x 20 images were captured from the perimeter of each tumor in Aperio ImageScope v11.1.2.760 software. The average number of F4/80 positive macrophages per field of view for each tumor was determined by counting manually. The amount of Ki67 labelling (termed positivity) was quantified from the intensity of labelling in individual tumors using the positive pixel algorithm in Aperio ImageScope software. In this algorithm, the number of pixels generated from the Ki67 staining was normalized to the total number of pixels generated from the haematoxylin counter stain. CD31 staining was quantified using a chalkley grid graticule and a 20x objective (Leica DMI4000, Germany). The grid was placed over five randomly selected fields of view within each site of early or late carcinoma and aligned so that the maximum number of the 25 dots were superimposed over the CD31 staining [19]. The sum value of the scores generated for each tumor was termed the Cumulative Chalkey Score (CCS) as a measure of microvessel density (MVD).

## Statistics

All data analysis was carried out in IBM SPSS Statistics for Windows (Version 22.0) or Graphpad Prism (Version 7.0b). The mean number of F4/80 positive liver macrophages per section (the dependent variable) was analysed by univariate analysis taking into account treatment group and diet (fixed factors) and animal ID (random factor) with Tukey post hoc tests to identify any differences between treatment groups. In analyzing data from sections of mammary fat pad and tumor we were consistent with previous studies using the PyMT model by including data from individual tumors rather than individual mice [18, 20, 21]. Data were divided into four treatment groups based on the combination of genotype and diet (PyMT control diet, PyMT doxy diet, PyMT-MacLow control diet, and PyMT-MacLow doxy diet). To avoid falsely significant results we carried out a nested analysis consistent with previous tumor based studies [22–25] taking into account which mouse each tumor was from and which treatment groups each mouse belonged to. This analysis was carried out for the grouped data of all the tumors from each treatment group and repeated with data divided according to tumor grade. Results are presented in graphs as single data points representing the data from each tumor. Error bars represent ± standard deviation. A Chi-square test was carried out to compare the percentage of tumors at the hyperplasia or higher grade.

## Results

### CD68^+^ and F4/80^+^ macrophages are analogous populations in PyMT tumors

**Fig 1:**
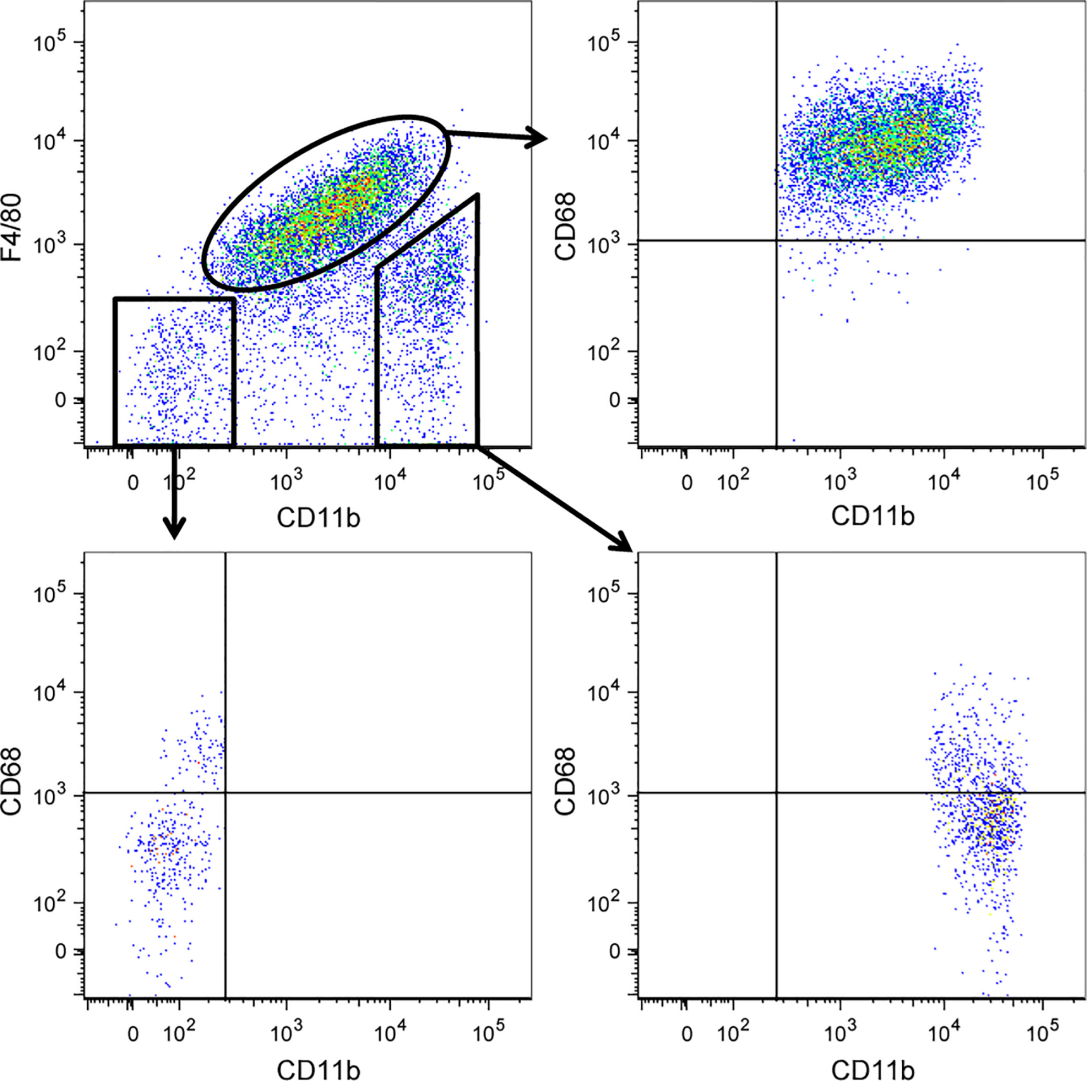
CD68+ and F4/80+ macrophages are analogous populations in PyMT tumors. Tumors from PyMT mice were enzymatically digested and made into a single cell suspension. Cells were stained with antibodies against the surface markers CD45, CD11b and F4/80. Cells were then fixed, permeabilized and stained with anti-CD68. Fluorescence was measured by flow cytometry and the data was analyzed using Flowjo software. Dot plots shown were generated from CD45^+^ cells. CD68 expression was determined after gating on tumor-associated macrophages (CD45^+^CD11b^+^F4/80^+^), CD11b^+^F4/80^—^cells and CD11b^—^F4/80^—^cells. Representative dot plots are shown from one of four mice analysed.

F4/80 is the most commonly used marker to identify TAMs in mouse tissue. To understand the extent of overlap between F4/80- and CD68-expressing macrophages in MMTV-PyMT tumors, we performed flow cytometry on dissociated tumor tissue. After gating on CD45^+^ immune cells, we plotted CD11b versus F4/80 and gated on CD11b^+^F4/80^+^ TAMs. We found that nearly 100% of CD11b^+^F4/80^+^ TAMs express CD68 (**Fig 1**). Some CD11b^+^F4/80^—^cells, which include monocytes, neutrophils and other myeloid cells, expressed lower levels of CD68 and these cells are likely newly recruited monocytes differentiating into TAMs. Not surprisingly, a small proportion of CD11b^—^F4/80^—^also expressed CD68 (**Fig 1**), as a proportion of CD11b^—^CD11c^+^CD103^+^ dendritic cells are known to express CD68 in various tissues [26, 27]. These data indicate that F4/80 is a marker of CD68^+^ TAMs in MMTV-PyMT mice.

### Doxycycline treatment decreases macrophage numbers in PyMT-MacLow mice

We crossed the MMTV-PyMT mammary tumor model [15] with MacLow mice [14] to generate PyMT-MacLow mice. To determine whether doxycycline treatment had the same effect on macrophage numbers in distant organs of tumor-bearing PyMT-MacLow as was previously observed in the tumor-free MacLow mouse model [14], sections of liver from treated and untreated MacLow, tumor-bearing PyMT and tumor-bearing PyMT-MacLow mice were immunohistochemically labelled for F4/80. Doxycycline treatment reduced the number of macrophages in the liver by 48% in MacLow animals and 41% in tumor-bearing PyMT-MacLow animals when compared to untreated mice (**S1 Fig**). The level of macrophage depletion observed in the liver following doxycycline treatment of tumor-bearing PyMT-MacLow and tumor-free MacLow animals was consistent with our previous findings where 6 weeks of doxycycline treatment resulted in ~50% reduction in the number of macrophages in samples of liver, spleen and bone [14]. As expected, the number of macrophages in tumor-bearing PyMT animals was not affected by doxycycline (**S1 Fig**).

**Fig 2:**
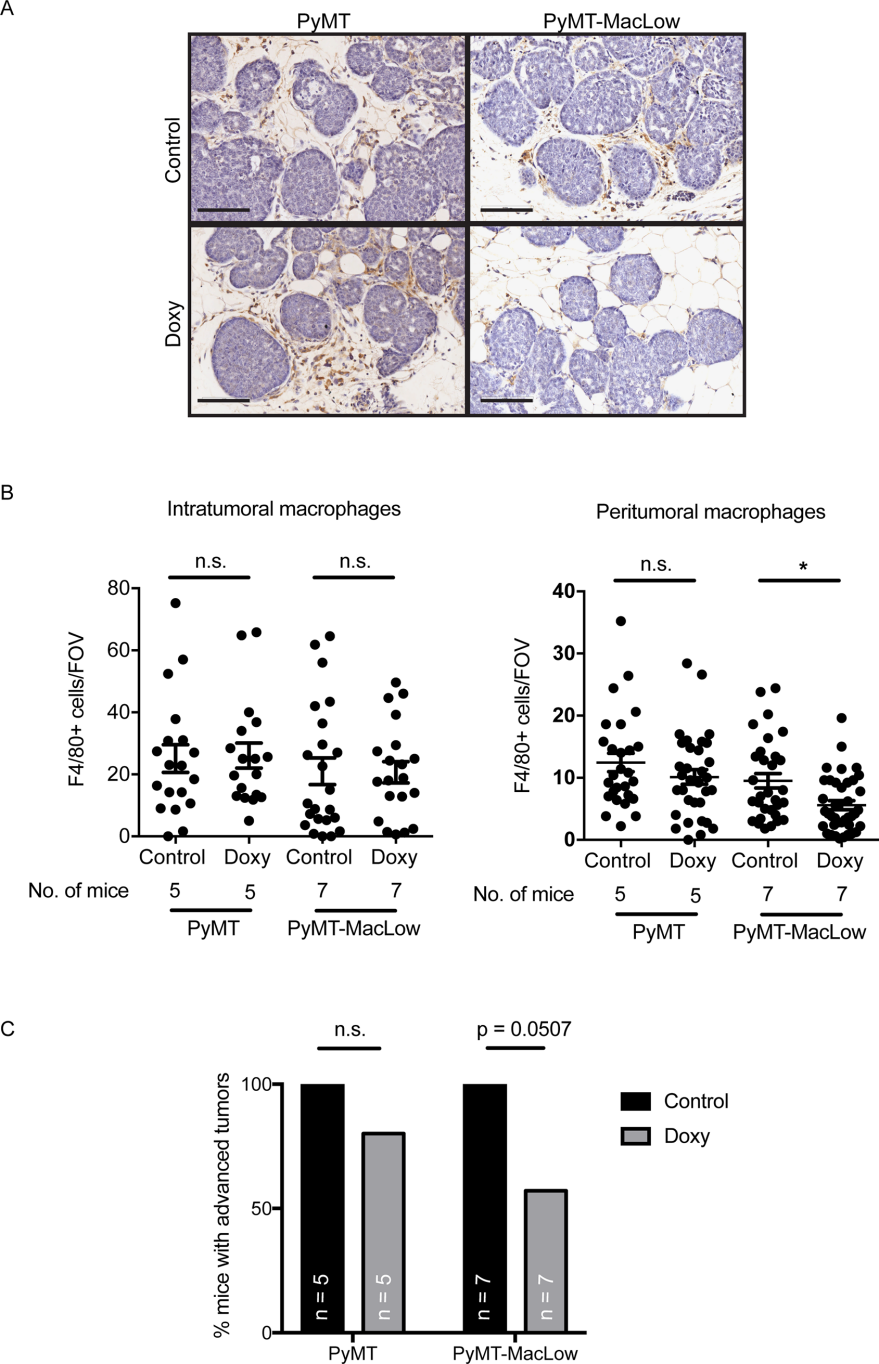
Doxycycline reduces the number of macrophages surrounding mammary tumors in PyMT-MacLow mice. (**A**) Sections of mammary tissue were labelled for F4/80 (DAB brown cells) and counterstained with haematoxylin; a representative image at the early carcinoma stage of tumor development is shown for each genotype and treatment. (**B**) Images captured from slides scanned on an Aperio slide scanner were used to quantify the number of macrophages within (intratumoral) and on the perimeter of mammary tumors (peritumoral). The data was then grouped according to genotype and treatment group and the mean value +/- SD shown. Each individual data point represent the mean values for an individual tumor. Tumor samples were obtained from the following numbers of animals per treatment group the number of tumors analysed is indicated in brackets: PyMT UT = 5(27), PyMT Doxy = 5(33), PyMT-MacLow UT = 7(31), PyMT-MacLow Doxy = 7(39). Significance is from the SPSS nested analysis comparing data from doxycycline treated versus control animals for each tumor and genotype. NS = not significant, **P<0.01. Scalebar = 100 μm. (C) The percentage of animals that had any tumors of hyperplasia and/or Adenoma/mammary intraepithelial neoplasia stages (Adenoma/MIN) was calculated for each genotype and treatment group. A Chi-square test was used to calculate statistical significance.

To address the main aim of this study, we assessed the number of macrophages in tumors from PyMT and PyMT-MacLow mice treated with and without doxycycline. F4/80^+^ TAMs were quantified in intratumoral and peritumoral areas (**Fig 2A**). Following doxycycline treatment, there was no change in the mean number of intratumoral macrophages in either tumor-bearing PyMT or PyMT-MacLow animals (**Fig 2B**). However, peritumoral macrophages were significantly reduced by 43% after doxycycline treatment of tumor-bearing PyMT-MacLow mice (**Fig 2B**). These data indicate that doxycycline-induced depletion of CD68-expressing cells largely affects peritumoral macrophages.

### Tumor progression is delayed in macrophage-deficient PyMT-MacLow animals

We then examined the rate of tumor formation in PyMT-MacLow mice. Tumor latency and multiplicity was unchanged between untreated PyMT and PyMT-MacLow mice (data not shown). Tumor latency and multiplicity was also equal between untreated and doxycycline-treated PyMT-MacLow mice (data not shown). Next, we investigated how the depletion of macrophages influenced tumor stage in PyMT-MacLow mice. The PyMT mammary tumor model follows a stepwise progression of disease: from hyperplasia to late carcinoma [15]. At 10 weeks of age, both PyMT and PyMT-MacLow mice had multiple tumors of different grades, consistent with the phenotype of the model (**S1 Table and Fig 2C**). The percentage of animals that had tumors of Adenoma/MIN or a higher stage was calculated for each genotype and treatment group (**Fig 2C**). A striking difference between macrophage-deficient animals (doxycycline-treated PyMT-MacLow mice) and control animals was the observation that far fewer PyMT-Maclow animals treated with doxycycline (4/7, 57%) had carcinomas than either control PyMT-Maclow (7/7, 100%) or treated PyMT animals (4/5, 80%). In fact 3 out of 7 doxycycline treated animals did not possess any tumors higher than the hyperplasia stage (**S1 Table)** whereas doxycycline treatment of PyMT mice did not significantly affect the staging of tumors (**Fig 2C and S1 Table**) where only 1/5 animals did not possess any tumours above the hyperplasia stage. Thus, depletion of peri-tumoral TAMs correlates with a delay in tumor progression.

### The proliferative capacity of tumors is reduced in macrophage deficient animals

**Fig 3:**
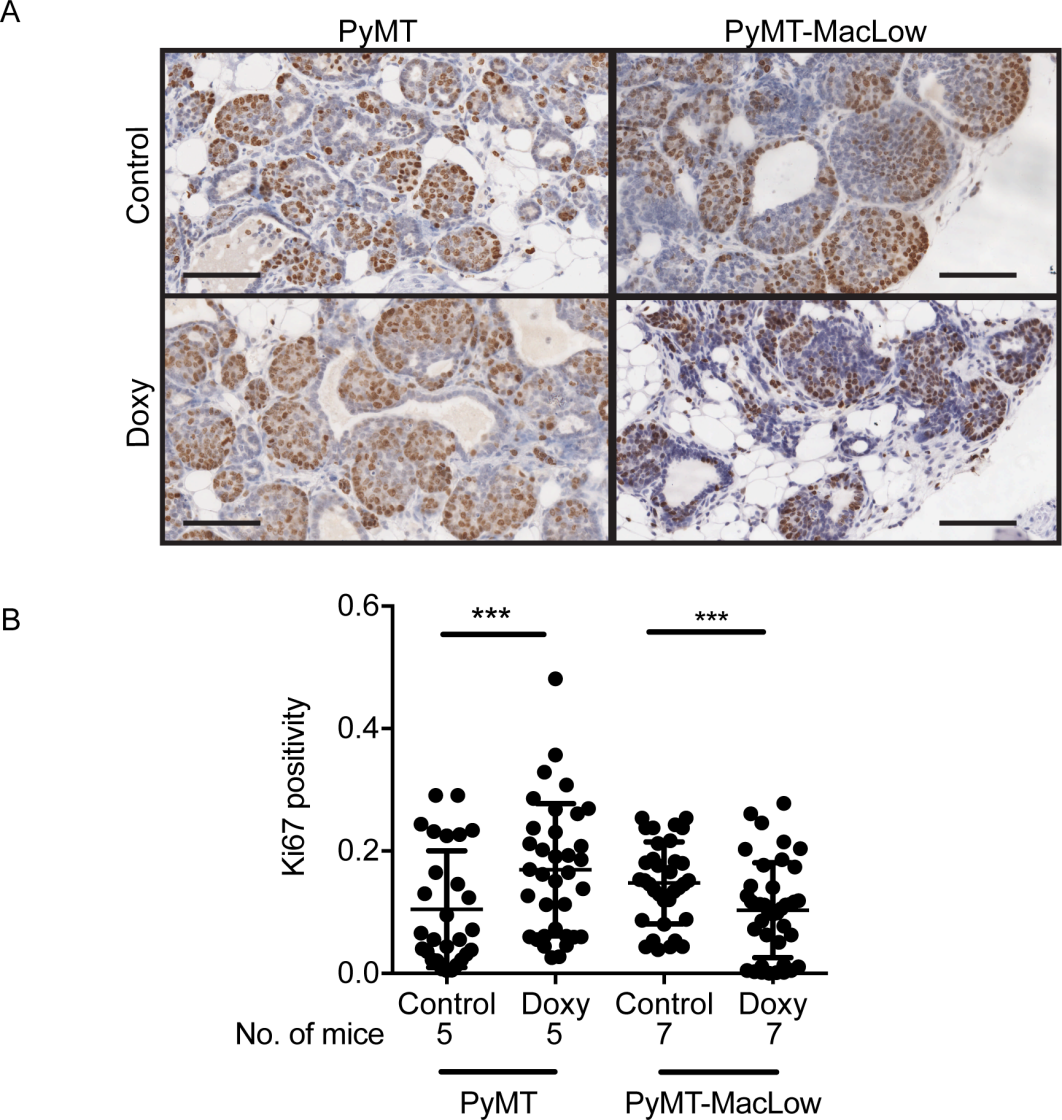
The proliferative capacity of Adenoma/MIN tumors is reduced in macrophage deficient mice. Mammary sections were labelled with an anti-Ki67 antibody as a marker of proliferation and counterstained with haematoxylin. (**A**) Images were captured from slides scanned on an Aperio slide scanner and split according to tumor grade, a representative image of an Adeno/MIN tumor (A/M) is shown for each genotype and treatment group. (**B**) The amount of Ki67 labelling in individual tumor areas (cells stained dark brown with DAB) was quantified and expressed as positivity using the Aperio positive pixel algorithm. Each data point on the graph represents the mean positivity for an individual tumor and the number of animals these tumors were taken from is shown below the x axis on each graph. The mean value ± SD was plotted and a nested analysis carried out in SPSS to compare data from doxycycline treated versus control animals from each tumor grade and genotype. ***P<0.001. Scalebar = 100 μm.

To gain insight into how macrophages promote cancer progression, tumor sections were labelled for the proliferation marker Ki67 (**Fig 3A**). Surprisingly, Ki67 positivity in PyMT tumors was increased by doxycycline treatment (**Fig 3B**), suggesting that doxycycline positively affects cancer cell proliferation or Ki67 expression. When comparing untreated and doxycycline-treated PyMT-MacLow mice, Ki67 positivity was markedly reduced in TAM-deficient tumors (**Fig 3B**). These data demonstrate that TAMs positively regulate the proliferative capacity of PyMT tumors.

### Angiogenesis is impaired in carcinomas from macrophage-deficient PyMT-MacLow mice

**Fig 4:**
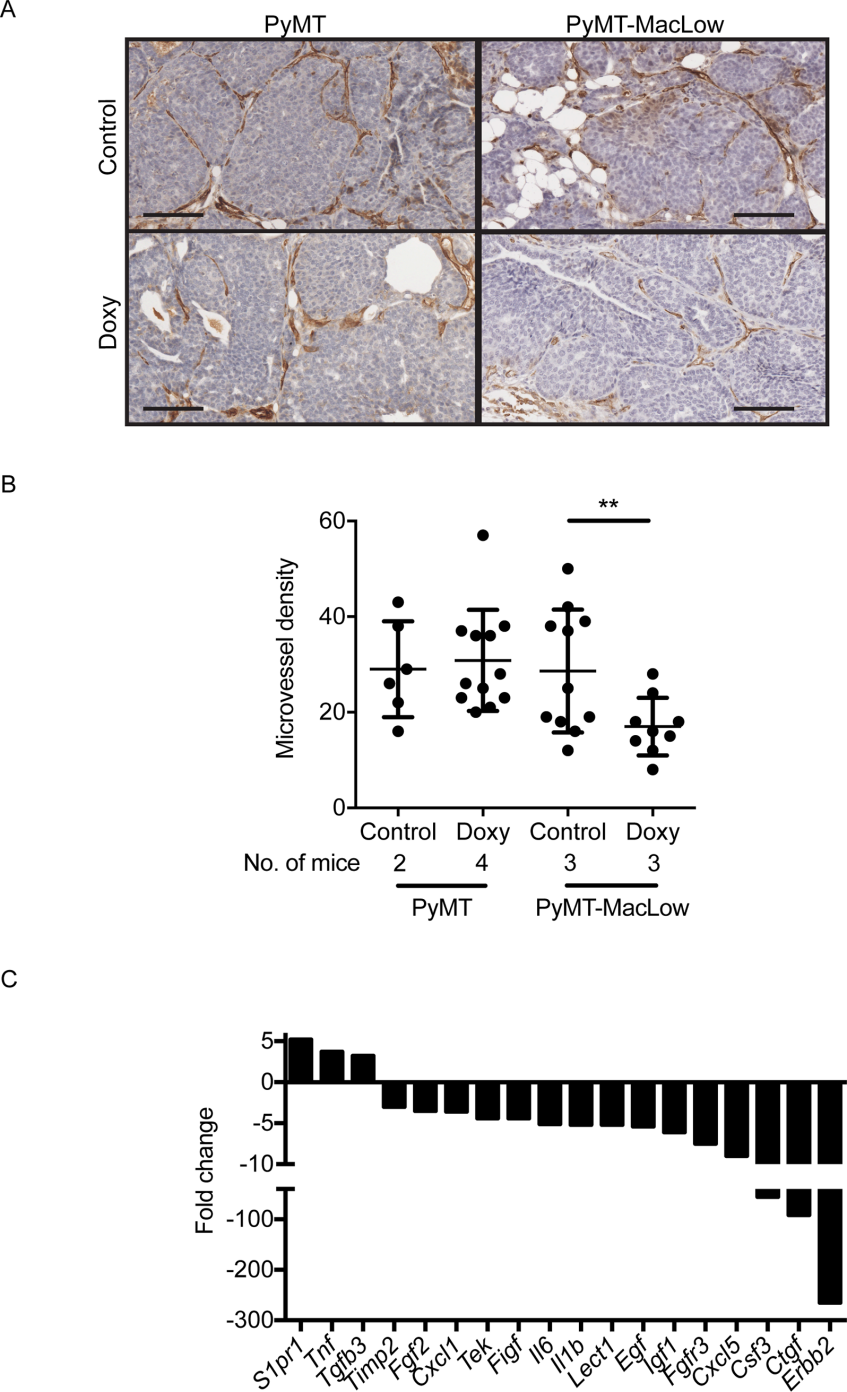
Loss of CD68^+^ macrophages negatively affects angiogenesis in PyMT mice. Mammary sections were labelled with rabbit anti-mouse antibodies against CD31 (DAB brown) a marker of angiogenesis and counterstained with haematoxylin. (**A**) Images were captured from slides scanned on an Aperio slide scanner and a representative image of an early carcinoma is shown for both treatment groups. (**B**) Microvessel density (MVD) was expressed as cumulative chalkley score (CCS). Each point on the graph represents the CCS for individual early and late carcinomas obtained from the following numbers of animals: PyMT UT = 2(6), PyMT Doxy = 4(12), PyMT-MacLow UT = 3(11), PyMT-MacLow Doxy = 3(9), numbers in brackets correspond to the total number of carcinomas analysed for each group, the number of animals is also displayed on the graph below the axis. (**C**) Three samples of cDNA from mammary fat pads containing tumors were pooled for control and doxycycline treated PyMT-MacLow animals. Gene expression levels in the pooled cDNA samples were determined by hybridisation to two Mouse Angiogenesis RT^2^ (Qiagen) plates (control and treated). Data was generated and interpreted in SDS 2.3 and any genes exhibiting a three-fold or greater change in the treated versus control group were considered biologically relevant and are shown on the graph. Scalebar = 100 μm. For part B nested analysis of the data was carried out in SPSS, **P<0.01.

Macrophages regulate the angiogenic switch in PyMT tumors [10]. To determine whether depletion of CD68^+^ TAMs influenced angiogenesis, tumor sections from untreated and doxycycline-treated PyMT-MacLow mice were immunohistochemically labelled using antibodies to the endothelial cell marker CD31 (**Fig 4A**). This analysis revealed that microvessel density is decreased in TAM-deficient tumors and tumors lacking TAMs displayed a 36% reduction in CD31^+^ blood vessels when compared with TAM-proficient tumors (**Fig 4B**). We then used an angiogenesis-focused, PCR-based array to investigate expression of angiogenesis-associated genes. We observed a decrease in several pro-angiogenic genes, including, but not limited to *Timp2*, *Fgf2*, *Tek* (which encodes the angiopoietin receptor, TIE2), *Il6*, *Egf*, *Igf1* and *Fgfr3* (**Fig 4C**). These data demonstrate that deficiency of CD68^+^ TAMs negatively affects angiogenesis in PyMT-MacLow tumors.

## Discussion

The fields of cancer biology and immunology lack sophisticated genetic models that allow the inducible and selective deletion of macrophages to study the role of these cells in tumor progression, metastasis and anti-cancer therapy treatment. As such, we generated the PyMT-MacLow model. Here, we show using the PyMT-MacLow model that depleting CD68^+^ macrophages in a controlled, specific and timely manner negatively affects the development of mammary ductal adenocarcinoma. Our model enabled the depletion of macrophages without compromising other aspects of tissue homeostasis and stands in contrast to the *Csf1*^*op*^*/Csf1*^*op*^ mouse where the total lack of macrophages causes impaired mammary gland development [28], growth retardation, infertility and impaired pancreatic development [29, 30]. Due to these confounding phenotypes, the aspects of ill-health associated with the *Csf1*^*op*^*/Csf1*^*op*^ mouse and other mouse models of macrophage ablation are not ideal for studying disease processes. The MacLow and PyMT-MacLow models overcome many of these limitations [14].

Consistent with the previously published pro-tumorigenic role of TAMs in the MMTV-PyMT model [9, 10], doxycycline-induced macrophage depletion reduced the proliferative capacity and angiogenic potential of PyMT tumors. Previous studies from various labs have shown that TAMs stimulate the angiogenic switch in PyMT tumors, which allows tumors to transition to malignancy [9, 10]. Subsets of TAMs, particularly those that express TIE2, are potent effectors of angiogenesis [31, 32], as demonstrated by specific deletion of the TIE2-expressing TAM population [31] or knockdown of *Tek/Tie2* mRNA in TAMs [33]. TIE2-expressing TAMs produce FGF2 that can activate endothelial cells [31, 32]. In line with these data, we found that *Fgf2* and *Tek/Tie2* mRNA are decreased in macrophage-deficient PyMT-MacLow tumors. This observation supports the use of the PyMT-MacLow model for angiogenesis research.

TAMs not only influence endothelial cells in the MMTV-PyMT model, but cancer cells as well. The crosstalk between TAMs and cancer cells is required for invasion and metastasis, whereby cancer cells provide CSF1 for TAMs, and in turn TAMs produce EGF that feeds back on cancer cells to stimulate their movement [34, 35]. In addition to CSF1, IL-4 from CD4^+^ T cells is necessary to activate TAMs to produce EGF and without CD4^+^ T cells, TAMs are unable to induce cancer cell migration and metastasis [36]. As further evidence that the PyMT-MacLow model accords well with this mechanism, we found that *Egf* mRNA levels are reduced in macrophage-deficient mice. Intravital imaging has shown that cancer cell intravasation into the circulation occurs at the invasive front of tumors where macrophages are highly abundant [37]. Thus, the PyMT-MacLow model may be particularly important for invasion and metastasis studies, as peri-tumoral macrophages are preferentially depleted in this model. Moreover, questions regarding the functional differences between peri-tumoral and intra-tumoral macrophages can be answered with the PyMT-MacLow model by depleting peri-tumoral populations while leaving intra-tumoral populations intact. The PyMT-MacLow will be also useful to further investigate TAM-cytotoxic T cell interactions at tumor margins, where the two cell types co-localize in PyMT tumors [38, 39].

## Conclusions

In the current study, we established the PyMT-MacLow model as novel tool to study the role of macrophages during breast cancer progression. The inducible and specific depletion of CD68^+^ cells offers flexibility to investigate the role of TAMs at different stages of tumor development, growth, metastasis and during anti-cancer therapy treatment. The PyMT-MacLow model is devoid of the side effects observed in previous depletion and knockout models. Having reproduced many phenotypes from previously published reports, the PyMT-MacLow will be a valuable asset to the macrophage and cancer biology communities.

## Supporting information

S1 ARRIVE Checklist (PDF)

S1 Fig Doxycycline treatment decreases macrophage numbers in the livers of PyMT-MacLow animals. MacLow, PyMT and PyMT-MacLow mice were treated with doxycycline for seven weeks and the liver harvested. Tissue from untreated mice and doxycycline treated PyMT mice were all used as negative controls. (**A**) Sections of liver were labelled with rat anti-mouse F4/80 antibody and counterstained with haematoxylin, F4/80 positive cells stain dark brown with DAB. (**B**) The number of macrophages remaining after doxycycline administration counted from positively stained cells in five fields of view per animal at 20 x magnification. The data is represented as the average number of cells per field of view +/- standard deviation (SD). The number of animals per group is indicated on the graph below the x axis. Data was analysed by Univariate Analysis in SPSS (Version 22.0) to compare data from doxycycline treated (Doxy) versus untreated (UT) control animals for each genotype. ***P<0.0001. Scalebar = 50 μm.
(PDF)

S1 Table Tumor information for each animal. The number of tumors at each stage of disease is detailed for all animals studied where each row corresponds to individual untreated (first box) and doxycycline treated (second box) animals. The 3/7 doxycycline treated PyMT-MacLow animals and the 1/5 doxycycline treated PyMT animal that did not possess any tumors higher than hyperplasia stage are highlighted yellow and green respectively. n = 5 for control and treated PyMT, n = 7 for control and treated PyMT-MacLow.
(DOCX)

## Abbreviations

A/MIN: Adenoma/mammary intraepithelial neoplasia
doxy: doxycycline; EC: early carcinoma
H: hyperplasia
LC: late carcinoma
MacLow: macrophage Low
MCP-1: monocyte chemoattractant protein-1
MMTV: mouse mammary tumor virus
MVD: microvessel density
PyMT: polyoma virus middle T-antigen
TAM: tumor associated macrophage
UT: untreated (animals on control diet)

## References

[1] Wynn TA, Chawla A, Pollard JW. Macrophage biology in development, homeostasis and disease. Nature. 2013;496(7446):445–55. doi: 10.1038/nature12034 ; PubMed Central PMCID: PMCPMC3725458. 23619691 10.1038/nature12034 PMC3725458

[2] Ostuni R, Kratochvill F, Murray PJ, Natoli G. Macrophages and cancer: from mechanisms to therapeutic implications. Trends Immunol. 2015;36(4):229–39. doi: 10.1016/j.it.2015.02.004 . 25770924 10.1016/j.it.2015.02.004

[3] Ruffell B, Coussens LM. Macrophages and therapeutic resistance in cancer. Cancer Cell. 2015;27(4):462–72. doi: 10.1016/j.ccell.2015.02.015 ; PubMed Central PMCID: PMC4400235. 25858805 10.1016/j.ccell.2015.02.015 PMC4400235

[4] Noy R, Pollard JW. Tumor-associated macrophages: from mechanisms to therapy. Immunity. 2014;41(1):49–61. Epub 2014/07/19. doi: 10.1016/j.immuni.2014.06.010 ; PubMed Central PMCID: PMC4137410. 25035953 10.1016/j.immuni.2014.06.010 PMC4137410

[5] De Palma M, Lewis CE. Macrophage regulation of tumor responses to anticancer therapies. Cancer Cell. 2013;23(3):277–86. Epub 2013/03/23. doi: 10.1016/j.ccr.2013.02.013 . 23518347 10.1016/j.ccr.2013.02.013

[6] Coffelt SB, de Visser KE. Immune-mediated mechanisms influencing the efficacy of anticancer therapies. Trends Immunol. 2015;36(4):198–216. doi: 10.1016/j.it.2015.02.006 . 25857662 10.1016/j.it.2015.02.006

[7] Ries CH, Cannarile MA, Hoves S, Benz J, Wartha K, Runza V, et al Targeting tumor-associated macrophages with anti-CSF-1R antibody reveals a strategy for cancer therapy. Cancer Cell. 2014;25(6):846–59. Epub 2014/06/06. doi: 10.1016/j.ccr.2014.05.016 . 24898549 10.1016/j.ccr.2014.05.016

[8] Butowski N, Colman H, De Groot JF, Omuro AM, Nayak L, Wen PY, et al Orally administered colony stimulating factor 1 receptor inhibitor PLX3397 in recurrent glioblastoma: an Ivy Foundation Early Phase Clinical Trials Consortium phase II study. Neuro Oncol. 2016;18(4):557–64. doi: 10.1093/neuonc/nov245 ; PubMed Central PMCID: PMCPMC4799682. 26449250 10.1093/neuonc/nov245 PMC4799682

[9] Lin EY, Nguyen AV, Russell RG, Pollard JW. Colony-stimulating factor 1 promotes progression of mammary tumors to malignancy. J Exp Med. 2001;193(6):727–40. . 11257139 10.1084/jem.193.6.727 PMC2193412

[10] Lin EY, Li JF, Gnatovskiy L, Deng Y, Zhu L, Grzesik DA, et al Macrophages regulate the angiogenic switch in a mouse model of breast cancer. Cancer Res. 2006;66(23):11238–46. doi: 10.1158/0008-5472.CAN-06-1278 . 17114237 10.1158/0008-5472.CAN-06-1278

[11] Galmbacher K, Heisig M, Hotz C, Wischhusen J, Galmiche A, Bergmann B, et al Shigella mediated depletion of macrophages in a murine breast cancer model is associated with tumor regression. PloS one. 2010;5(3):e9572 doi: 10.1371/journal.pone.0009572 ; PubMed Central PMCID: PMC2833200. 20221397 10.1371/journal.pone.0009572 PMC2833200

[12] Lewen S, Zhou H, Hu HD, Cheng T, Markowitz D, Reisfeld RA, et al A Legumain-based minigene vaccine targets the tumor stroma and suppresses breast cancer growth and angiogenesis. Cancer Immunol Immunother. 2008;57(4):507–15. doi: 10.1007/s00262-007-0389-x . 17786443 10.1007/s00262-007-0389-x PMC11030723

[13] Zeisberger SM, Odermatt B, Marty C, Zehnder-Fjallman AH, Ballmer-Hofer K, Schwendener RA. Clodronate-liposome-mediated depletion of tumour-associated macrophages: a new and highly effective antiangiogenic therapy approach. Br J Cancer. 2006;95(3):272–81. doi: 10.1038/sj.bjc.6603240 . 16832418 10.1038/sj.bjc.6603240 PMC2360657

[14] Gheryani N, Coffelt SB, Gartland A, Rumney RM, Kiss-Toth E, Lewis CE, et al Generation of a novel mouse model for the inducible depletion of macrophages in vivo. Genesis. 2013;51(1):41–9. Epub 2012/08/29. doi: 10.1002/dvg.22343 . 22927121 10.1002/dvg.22343

[15] Guy CT, Cardiff RD, Muller WJ. Induction of mammary tumors by expression of polyomavirus middle T oncogene: a transgenic mouse model for metastatic disease. Mol Cell Biol. 1992;12(3):954–61. Epub 1992/03/01. . 1312220 10.1128/mcb.12.3.954-961.1992 PMC369527

[16] Lin EY, Jones JG, Li P, Zhu L, Whitney KD, Muller WJ, et al Progression to malignancy in the polyoma middle T oncoprotein mouse breast cancer model provides a reliable model for human diseases. Am J Pathol. 2003;163(5):2113–26. doi: 10.1016/S0002-9440(10)63568-7 ; PubMed Central PMCID: PMC1892434. 14578209 10.1016/S0002-9440(10)63568-7 PMC1892434

[17] Coffelt SB, Kersten K, Doornebal CW, Weiden J, Vrijland K, Hau CS, et al IL-17-producing gammadelta T cells and neutrophils conspire to promote breast cancer metastasis. Nature. 2015;522(7556):345–8. doi: 10.1038/nature14282 ; PubMed Central PMCID: PMCPMC4475637. 25822788 10.1038/nature14282 PMC4475637

[18] Ottewell PD, Brown HK, Jones M, Rogers TL, Cross SS, Brown NJ, et al Combination therapy inhibits development and progression of mammary tumours in immunocompetent mice. Breast cancer research and treatment. 2012;133(2):523–36. doi: 10.1007/s10549-011-1782-x . 21956211 10.1007/s10549-011-1782-x

[19] Bluff JE, Menakuru SR, Cross SS, Higham SE, Balasubramanian SP, Brown NJ, et al Angiogenesis is associated with the onset of hyperplasia in human ductal breast disease. Br J Cancer. 2009;101(4):666–72. doi: 10.1038/sj.bjc.6605196 ; PubMed Central PMCID: PMC2736809. 19623180 10.1038/sj.bjc.6605196 PMC2736809

[20] Lunt SJ, Kalliomaki TM, Brown A, Yang VX, Milosevic M, Hill RP. Interstitial fluid pressure, vascularity and metastasis in ectopic, orthotopic and spontaneous tumours. BMC Cancer. 2008;8:2 doi: 10.1186/1471-2407-8-2 ; PubMed Central PMCID: PMC2245966. 18179711 10.1186/1471-2407-8-2 PMC2245966

[21] Ricciardelli C, Frewin KM, Tan Ide A, Williams ED, Opeskin K, Pritchard MA, et al The ADAMTS1 protease gene is required for mammary tumor growth and metastasis. Am J Pathol. 2011;179(6):3075–85. doi: 10.1016/j.ajpath.2011.08.021 ; PubMed Central PMCID: PMC3260838. 22001177 10.1016/j.ajpath.2011.08.021 PMC3260838

[22] Mahmud SM, Franco EL, Turner D, Platt RW, Beck P, Skarsgard D, et al Use of non-steroidal anti-inflammatory drugs and prostate cancer risk: a population-based nested case-control study. PloS one. 2011;6(1):e16412 doi: 10.1371/journal.pone.0016412 ; PubMed Central PMCID: PMC3030588. 21297996 10.1371/journal.pone.0016412 PMC3030588

[23] Charlton RA, Snowball JM, Bloomfield K, de Vries CS. Colorectal cancer risk reduction following macrogol exposure: a cohort and nested case control study in the UK. PloS one. 2013;8(12):e83203 doi: 10.1371/journal.pone.0083203 ; PubMed Central PMCID: PMC3869778. 24376663 10.1371/journal.pone.0083203 PMC3869778

[24] Yang JJ, Cho LY, Ma SH, Ko KP, Shin A, Choi BY, et al Oncogenic CagA promotes gastric cancer risk via activating ERK signaling pathways: a nested case-control study. PloS one. 2011;6(6):e21155 doi: 10.1371/journal.pone.0021155 ; PubMed Central PMCID: PMC3116873. 21698158 10.1371/journal.pone.0021155 PMC3116873

[25] Wright E, Schofield PT, Seed P, Molokhia M. Bisphosphonates and risk of upper gastrointestinal cancer—a case control study using the General Practice Research Database (GPRD). PloS one. 2012;7(10):e47616 doi: 10.1371/journal.pone.0047616 ; PubMed Central PMCID: PMC3480418. 23112825 10.1371/journal.pone.0047616 PMC3480418

[26] Zaynagetdinov R, Sherrill TP, Kendall PL, Segal BH, Weller KP, Tighe RM, et al Identification of myeloid cell subsets in murine lungs using flow cytometry. Am J Respir Cell Mol Biol. 2013;49(2):180–9. doi: 10.1165/rcmb.2012-0366MA ; PubMed Central PMCID: PMCPMC3824033. 23492192 10.1165/rcmb.2012-0366MA PMC3824033

[27] Tamoutounour S, Guilliams M, Montanana Sanchis F, Liu H, Terhorst D, Malosse C, et al Origins and functional specialization of macrophages and of conventional and monocyte-derived dendritic cells in mouse skin. Immunity. 2013;39(5):925–38. doi: 10.1016/j.immuni.2013.10.004 . 24184057 10.1016/j.immuni.2013.10.004

[28] Pollard JW, Hennighausen L. Colony stimulating factor 1 is required for mammary gland development during pregnancy. Proc Natl Acad Sci U S A. 1994;91(20):9312–6. ; PubMed Central PMCID: PMC44802. 7937762 10.1073/pnas.91.20.9312 PMC44802

[29] Banaei-Bouchareb L, Gouon-Evans V, Samara-Boustani D, Castellotti MC, Czernichow P, Pollard JW, et al Insulin cell mass is altered in Csf1op/Csf1op macrophage-deficient mice. J Leukoc Biol. 2004;76(2):359–67. doi: 10.1189/jlb.1103591 . 15178709 10.1189/jlb.1103591

[30] Dai XM, Zong XH, Sylvestre V, Stanley ER. Incomplete restoration of colony-stimulating factor 1 (CSF-1) function in CSF-1-deficient Csf1op/Csf1op mice by transgenic expression of cell surface CSF-1. Blood. 2004;103(3):1114–23. doi: 10.1182/blood-2003-08-2739 . 14525772 10.1182/blood-2003-08-2739

[31] De Palma M, Venneri MA, Galli R, Sergi Sergi L, Politi LS, Sampaolesi M, et al Tie2 identifies a hematopoietic lineage of proangiogenic monocytes required for tumor vessel formation and a mesenchymal population of pericyte progenitors. Cancer Cell. 2005;8(3):211–26. doi: 10.1016/j.ccr.2005.08.002 . 16169466 10.1016/j.ccr.2005.08.002

[32] Venneri MA, De Palma M, Ponzoni M, Pucci F, Scielzo C, Zonari E, et al Identification of proangiogenic TIE2-expressing monocytes (TEMs) in human peripheral blood and cancer. Blood. 2007;109(12):5276–85. doi: 10.1182/blood-2006-10-053504 . 17327411 10.1182/blood-2006-10-053504

[33] Mazzieri R, Pucci F, Moi D, Zonari E, Ranghetti A, Berti A, et al Targeting the ANG2/TIE2 axis inhibits tumor growth and metastasis by impairing angiogenesis and disabling rebounds of proangiogenic myeloid cells. Cancer Cell. 2011;19(4):512–26. Epub 2011/04/13. doi: 10.1016/j.ccr.2011.02.005 . 21481792 10.1016/j.ccr.2011.02.005

[34] Wyckoff J, Wang W, Lin EY, Wang Y, Pixley F, Stanley ER, et al A paracrine loop between tumor cells and macrophages is required for tumor cell migration in mammary tumors. Cancer Res. 2004;64(19):7022–9. doi: 10.1158/0008-5472.CAN-04-1449 . 15466195 10.1158/0008-5472.CAN-04-1449

[35] Goswami S, Sahai E, Wyckoff JB, Cammer M, Cox D, Pixley FJ, et al Macrophages promote the invasion of breast carcinoma cells via a colony-stimulating factor-1/epidermal growth factor paracrine loop. Cancer Res. 2005;65(12):5278–83. doi: 10.1158/0008-5472.CAN-04-1853 . 15958574 10.1158/0008-5472.CAN-04-1853

[36] DeNardo DG, Barreto JB, Andreu P, Vasquez L, Tawfik D, Kolhatkar N, et al CD4(+) T cells regulate pulmonary metastasis of mammary carcinomas by enhancing protumor properties of macrophages. Cancer Cell. 2009;16(2):91–102. Epub 2009/08/04. S1535-6108(09)00216-5 [pii] doi: 10.1016/j.ccr.2009.06.018 . 19647220 10.1016/j.ccr.2009.06.018 PMC2778576

[37] Wyckoff JB, Wang Y, Lin EY, Li JF, Goswami S, Stanley ER, et al Direct visualization of macrophage-assisted tumor cell intravasation in mammary tumors. Cancer Res. 2007;67(6):2649–56. doi: 10.1158/0008-5472.CAN-06-1823 . 17363585 10.1158/0008-5472.CAN-06-1823

[38] Engelhardt JJ, Boldajipour B, Beemiller P, Pandurangi P, Sorensen C, Werb Z, et al Marginating dendritic cells of the tumor microenvironment cross-present tumor antigens and stably engage tumor-specific T cells. Cancer Cell. 2012;21(3):402–17. doi: 10.1016/j.ccr.2012.01.008 ; PubMed Central PMCID: PMC3311997. 22439936 10.1016/j.ccr.2012.01.008 PMC3311997

[39] Broz ML, Binnewies M, Boldajipour B, Nelson AE, Pollack JL, Erle DJ, et al Dissecting the tumor myeloid compartment reveals rare activating antigen-presenting cells critical for T cell immunity. Cancer Cell. 2014;26(5):638–52. doi: 10.1016/j.ccell.2014.09.007 ; PubMed Central PMCID: PMC4254577. 25446897 10.1016/j.ccell.2014.09.007 PMC4254577

