# Supplementary figures and images for: PyMT-Maclow: A novel, inducible, murine model for determining the role of CD68 positive cells in breast tumor development
Source: PLoS One. 2017 Dec 8;12(12):e0188591. doi: 10.1371/journal.pone.0188591 (PMC5722323; doi:10.1371/journal.pone.0188591)

Supplemental Figure 1

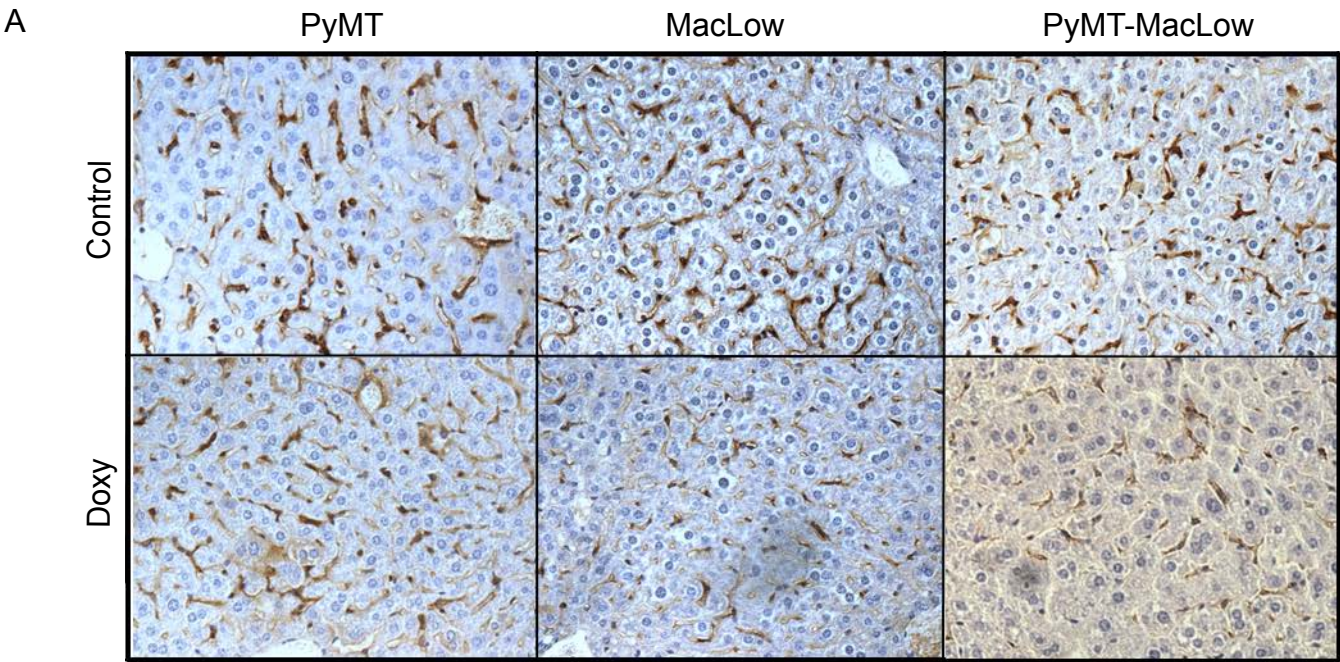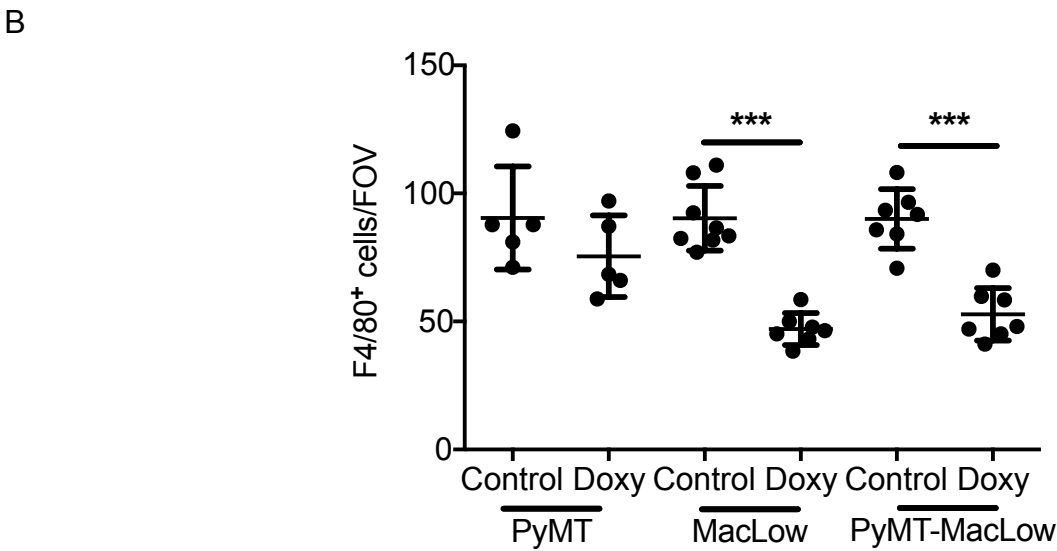

Supplement: S1 Fig — MacLow, PyMT and PyMT-MacLow mice were treated with doxycycline for seven weeks and the liver harvested. Tissue from untreated mice and doxycycline treated PyMT mice were all used as negative controls. (A) Sections of liver were labelled with rat anti-mouse F4/80 antibody and counterstained with haematoxylin, F4/80 positive cells stain dark brown with DAB. (B) The number of macrophages remaining after doxycycline administration counted from positively stained cells in five fields of view per animal at 20 x magnification. The data is represented as the average number of cells per field of view +/- standard deviation (SD). The number of animals per group is indicated on the graph below the x axis. Data was analysed by Univariate Analysis in SPSS (Version 22.0) to compare data from doxycycline treated (Doxy) versus untreated (UT) control animals for each genotype. ***P<0.0001. Scalebar = 50 μm. (PDF) [file pone.0188591.s002.pdf]
