# Supplementary material for: PyMT-Maclow: A novel, inducible, murine model for determining the role of CD68 positive cells in breast tumor development
Source: PLoS One. 2017 Dec 8;12(12):e0188591. doi: 10.1371/journal.pone.0188591 (PMC5722323; doi:10.1371/journal.pone.0188591)
Supplement: S1 Table — The number of tumors at each stage of disease is detailed for all animals studied where each row corresponds to individual untreated (first box) and doxycycline treated (second box) animals. The 3/7 doxycycline treated PyMT-MacLow animals and the 1/5 doxycycline treated PyMT animal that did not possess any tumors higher than hyperplasia stage are highlighted yellow and green respectively. n = 5 for control and treated PyMT, n = 7 for control and treated PyMT-MacLow. (DOCX) [file pone.0188591.s003.docx]

**S1 Table: Tumor information for each animal**

|  | No. of tumors | | | | | | | |
| --- | --- | --- | --- | --- | --- | --- | --- | --- |
|  | Untreated | | | | Doxycycline Treated | | | |
| Tumour Grade | H | A/M | EC | LC | H | A/M | EC | LC |
| PyMT-MacLow | 0 | 1 | 2 | 3 | 0 | 4 | 1 | 4 |
|  | 2 | 2 | 3 | 3 | 0 | 0 | 1 | 2 |
|  | 2 | 0 | 1 | 1 | 3 | 6 | 0 | 0 |
|  | 3 | 2 | 0 | 0 | 2 | 0 | 0 | 0 |
|  | 0 | 1 | 0 | 0 | 4 | 0 | 0 | 0 |
|  | 1 | 0 | 2 | 3 | 5 | 0 | 0 | 0 |
|  | 1 | 1 | 0 | 0 | 2 | 0 | 2 | 4 |
| PyMT | 1 | 2 | 3 | 4 | 0 | 2 | 2 | 3 |
|  | 0 | 0 | 1 | 0 | 1 | 0 | 1 | 0 |
|  | 2 | 3 | 1 | 1 | 4 | 0 | 1 | 3 |
|  | 1 | 0 | 1 | 0 | 6 | 0 | 0 | 0 |
|  | 3 | 1 | 0 | 4 | 4 | 1 | 4 | 3 |
